# Protocol for Minute Calisthenics: a randomized controlled study of a daily, habit-based, bodyweight resistance training program

**DOI:** 10.1186/s12889-020-09355-4

**Published:** 2020-08-15

**Authors:** Joshua C. Hollingsworth, Kaelin C. Young, Siraj F. Abdullah, Danielle D. Wadsworth, Ahmad Abukhader, Bari Elfenbein, Zachary Holley

**Affiliations:** 1 Edward Via College of Osteopathic Medicine - Auburn Campus, 910 South Donahue Drive, Auburn, AL 36832 USA; 2 Auburn University, School of Kinesiology, 301 Wire Road, Auburn, AL 36849 USA

**Keywords:** Resistance training, Exercise, Behavior change, Habit formation

## Abstract

**Background:**

Resistance-training (RT) provides significant health benefits. However, roughly 3/4 of adults in the United States do not meet current Physical Activity Guidelines in this regard. There has been a call for research examining the effectiveness of interventions to increase participation in physical activity and to better understand the dose response relationship upon health outcomes. Studies are needed that assess the effectiveness of RT programs that are time-efficient and simple to perform. This fully-powered, randomized controlled study will assess a habit-based RT program consisting of one set of push-ups, angled-rows, and bodyweight-squats performed every weekday for 12–24 weeks in untrained individuals.

**Methods:**

Forty–60 untrained osteopathic medical students and college/university employees who work in an office setting will be recruited and randomized (1:1) to an intervention or waitlist control group. After 12-week follow-up assessment, the intervention group will continue the program and the control group will initiate the program for 12 weeks. In addition to the equipment and training needed to safely perform the exercises, all participants will receive training in the Tiny Habits® Method (THM) and digital coaching for the duration of the study. Participants will complete weekly assessments regarding the program during their initial 12-week intervention phase. The primary outcome is the change from baseline to 12 weeks in the intervention group versus the control group, in the combined number of repetitions performed in one set of each of the three exercises (composite repetitions) under a standardized protocol. Secondary outcomes include adherence to and satisfaction with the program, and change from baseline to 12- and 24-week follow-up in blood pressure, fasting lipid panel, hemoglobin A1c, body mass index, anthropometry, body composition, mid-thigh muscle thickness, and habit strength.

**Discussion:**

This study will evaluate a simple, habit-based RT intervention in untrained individuals. The approach is unique in that it utilizes brief but frequent bodyweight exercises and, via the THM, focuses on consistency and habit formation first, with effort being increased as participants are motivated and able. If effective, the program can be easily scaled for wider adoption.

**Trial registration:**

This study was prospectively registered at ClinicalTrials.gov, identifier NCT04207567, on December 23rd, 2019.

## Background

There are many benefits to consistently engaging in resistance training (RT) over the long-term [1, 2]. Regular RT results in increased strength and muscle mass and decreased visceral fat, thereby improving body composition, metabolism, and physical performance [3]. In older populations, this translates into the prevention of sarcopenia and maintenance of functional strength [4]. In terms of losing weight, RT promotes “high quality” weight loss such that fat mass is lost while muscle mass is maintained or even increased [5]. RT has also been shown to reduce blood pressure [3], decrease low-density lipoprotein (LDL) cholesterol and triglycerides, and increase high-density lipoprotein (HDL) cholesterol [6, 7], all of which promotes cardiovascular health. In terms of the prevention and management of type 2 diabetes, RT has been shown to improve insulin sensitivity and reduce hemoglobin A1c (HbA1c) [8, 9]. In addition to the physical benefits, regular RT appears to improve cognitive functioning, particularly in older populations [10–12]. Overall, likely due to the combination of these and possibly other beneficial effects, regular RT and increased strength are associated with lower all-cause mortality [13, 14].

Current United States (U.S.) guidelines recommend that “muscle strengthening activities” of moderate-to-high intensity involving all the major muscle groups be performed on 2 or more days per week [2]. However, 74.5% of adults in the U.S. do not meet these guideline recommendations [15], and these estimates are lower still when specific activities such as RT are considered [16]. Even when individuals have good intentions [17] and the knowledge that there are significant benefits to be gained [18, 19], meaningful health behavior change can be difficult to initiate and to maintain over the long term. This includes health behaviors related to exercise and RT [20, 21]. Potential reasons for this difficulty include competing interests and responsibilities, actual or perceived lack of time, diminished motivation, forgetfulness or missed opportunities to act, lack of necessary training or ability, and lack of planning [20–22]. A simple program that utilizes the habit-formation process to incorporate brief bodyweight-based RT sessions into one’s weekday routine has the potential to address many of these barriers.

A habit, as traditionally  defined in behavioral psychology, is a behavior that is automatically prompted by a stable context cue that has been previously associated with the behavior through repetition and reward attainment [23, 24]. Examples include doing push-ups (behavior) after getting out of bed in the morning (cue) and going for a walk (behavior) after lunch (cue). The generally-accepted habit-formation process — consistently repeating a target behavior in response to a pre-identified context cue — is relatively simple [25, 26]. Before a habit is formed, behavioral control is greatly influenced by plans to take action, or lack thereof, which is why implementation intentions are initially helpful. However, as the habit develops, the behavior becomes less reliant on intentions and is instead prompted by environmental cues. Behavior automaticity increases and, at some point, peaks. The more automatic the behavior, the stronger the habit. Implementation intentions, which are plans specifying when and/or where a specific target behavior will be performed [27, 28], can be used to this end. Such plans make cues more mentally accessible and strengthen the cue-behavior association, thereby increasing perceptual readiness to act [29]. Implementation intentions have a medium-to-large effect on promoting engagement in various health behaviors, ranging from daily vitamin supplementation to self-breast examinations to exercise [30].

The Tiny Habits® Method (THM), created by BJ Fogg, PhD, is a systematic approach to habit formation that combines a specific form of implementation intention with simplification of the target behavior and immediate celebration of behavior performance. The implementation intentions of the THM are referred to as *Recipes*, which follow the format, “After I *Anchor moment*, I will *tiny Behavior*.” The *Anchor moment* is a reliable existing routine or event (e.g., After I get out of bed) that is used to prompt the new target behavior. The *tiny Behavior* (e.g., 1 push-up) is a simplified version of the target behavior (e.g., push-ups with High Effort). The individual can always do more, when they are motivated and able, but the focus is on always doing at least the *tiny Behavior* after the *Anchor moment.* This minimizes reliance on motivation and promotes consistency which, over time, increases the automaticity with which the *tiny Behavior* is performed. Each time a Recipe is completed successfully, a planned *Celebration* is performed. Anything that the individual can do in the moment to create positive emotions (e.g., do a fist-pump and say “Nailed it!”) may serve as the *Celebration*, the purpose of which is to provide an immediate reward that reinforces the new habit, further cultivating automaticity. Revision is part of the Tiny Habits process, whereby Recipes are revised as needed to find the best match for the individual [31].

Previous research suggests that a high-frequency, low-duration, bodyweight-based RT program would be effective in terms of increasing strength and improving body composition. Several studies have shown that bodyweight-based RT, such as push-ups approaching momentary muscular failure, performed consistently over 4–6 weeks can significantly increase muscle strength [32] and thickness [33, 34]. Similarly, other studies have shown that maximal isometric co-contraction, absent any external resistance, can elicit gains in strength and hypertrophy [35–38]. In terms of the relative amount of RT needed to increase strength, the current U.S. Physical Activity Guidelines acknowledge the fact that “one set... of each exercise is effective” when performed twice per week with significant effort [39]. However, evidence suggests a dose-response relationship, with higher training volume resulting in greater strength gains [40] and muscle hypertrophy [41]. Higher training volume can be achieved effectively by increasing the frequency of RT sessions [42–45]. Thus, engaging in brief bodyweight-based RT sessions every weekday is a means to increase RT frequency, and thus RT volume, while also facilitating habit formation [46, 47]. In terms of RT of the same muscle groups on consecutive days, a recent 12-week intervention study by Yang and colleagues [48] found that performing the same exercises on three consecutive days per week produced significant strength gains that were statistically no different than the strength gains realized from training on three non-consecutive days per week.

Although evidence suggests that there would be health benefits, a RT program similar to the one to be investigated here has not to our knowledge been systematically studied. The primary aim of this randomized controlled study is to implement and evaluate the effects of an RT program in untrained office workers and osteopathic medical students that utilizes brief bodyweight exercises and the THM. In addition to convenience, this population is being used for participant recruitment because their daily routine is generally stable and necessitates a significant amount of sedentary behavior on most weekdays. The primary exercises to be utilized include push-ups, angled-rows, and bodyweight-squats. These exercises require minimal equipment, can be modified to match most fitness levels and, together, target roughly 85% of skeletal muscle [49]. The THM is being utilized to promote consistency, habit formation, and overall adherence. The primary outcome of interest is the change in strength from baseline to 12-week follow-up, with the hypothesis that the change in strength will be greater in the intervention group relative to the change in the waitlist control group. Self-reported adherence to the program, satisfaction with the program, and habit strength will also be assessed, as will the change from baseline to follow-up in blood pressure, fasting lipid panel, HbA1c, body mass index (BMI), anthropometry, body composition, and mid-thigh muscle thickness, in all participants.

## Methods

This will be a 24-week, prospective, semi-crossover randomized controlled study with primary outcomes evaluated at 12 weeks. All procedures involved will be conducted in compliance with the U.S. Department of Health and Human Services (HHS) regulations regarding human subjects research. Ethical approval has been obtained from the Edward Via College of Osteopathic Medicine Institutional Review Board (VCOM IRB, local number 2019–037).

### Study population

A total of 40–60 office workers and osteopathic medical students will be recruited from a local college/university in Alabama and randomized (1:1) to the intervention group or waitlist control group using sealed envelopes, while controlling for sex via covariate adaptive randomization [50]. A power analysis was conducted to estimate sample size using the data published by Sperlich et al. [32] who conducted a similarly designed daily, body-weight-based, 6-min exercise intervention on changes in push-up performance and other health-based measures which resulted in a partial eta-squared effect size of 0.199 for the interaction effect for change in push-up performance. Using G*Power software (v. 3.1), an alpha level of 0.05, power of 0.80 and effect size (f) of 0.49 (determined using partial eta-square of 0.199), the estimated sample size needed is *n* = 34. Thus, our proposed sample size of *n* = 40–60 participants should be more than adequate to detect changes in our primary outcome while still allowing for some attrition. Email as well as digital and paper advertisements will be used to recruit participants.

In order to participate, the following inclusion/exclusion criteria must be met. Individuals must:

1. Be a college/university employee with their own office or cubicle, or an osteopathic medical student.
2. Be 19 years of age or older.
3. Indicate not being pregnant.
4. Indicate motivation and confidence in their ability to perform the exercises on most (4/5) weekdays.
5. Demonstrate the ability to properly perform at least 1 repetition of each of the 3 exercises: push-ups, angled-rows, and bodyweight-squats.
6. Indicate not having participated in structured RT for 2 or more days/week on average during the past year.
7. Have a location (e.g., a wall suitable for anchor placement or a suitable door at their home or office) to set up the suspension trainer needed to perform the angled-row exercise.
8. Indicate having no health issues that would significantly increase adverse event risk while participating, as assessed via the Physical Activity Readiness-Questionnaire [51].

### Enrollment and incentives

At the initial encounter, those who meet inclusion criteria will undergo informed consent and enrollment. All enrolled participants will then be assessed at baseline, 12-week follow-up, and 24-week follow-up. As a financial incentive, participants will receive a $50 Amazon gift card upon completion of the baseline and follow-up assessments, for a total of up to $150. If they complete all 3 assessments, then they can keep the exercise equipment (i.e., suspension trainer and plyometric box) that they were initially loaned for the study. Additionally, during the initial 12-weeks of their intervention phase, participants will be emailed a weekly assessment, and they will receive a $5 Amazon gift card for each weekly assessment that they complete, for a total of up to $60. Note that these financial incentives are not tied to exercise performance.

### Intervention

While participants randomized to the waitlist control group will be asked to refrain from RT for 12 weeks, participants randomized to the intervention group will be instructed to perform one “set” each of push-ups, angled-rows, and bodyweight-squats every weekday for 12 weeks. After the 12-week follow-up assessment for evaluation of primary outcomes, the intervention group will continue the RT program for an additional 12 weeks, and the control group will begin the RT program and continue for 12 weeks. Participants will perform the exercises individually and unsupervised in their home, school and/or office. In terms of preparation and guidance, all participants will receive the following prior to starting the intervention phase. They will be loaned a suspension trainer (Intent Sports®) and, if needed for office workers, have a wall anchor mounted in their office in order to perform the angled-row exercise. As a safety measure, they will also be loaned a 12”× 14”× 16” plyometric box (Synergee®), which they will place behind them and against the wall when performing bodyweight-squats. Participants will be instructed to report any adverse effects or harm directly to the principal investigator (PI), who will take appropriate action (e.g., remove participant(s) from study, if needed). Lastly, a one-on-one coach meeting with the PI will be scheduled.

The following is an outline of the coaching touchpoints and focus that will be utilized in the study. In the initial, one-on-one coach meeting, the participant will receive additional training regarding how to safely and properly perform each exercise, including variations to make each exercise easier or more difficult. They will also receive training on alternative exercises that can be performed, as needed (e.g., plank as alternative to push-ups if/when wrist pain is an issue, and wall-sit as an alternative to bodyweight-squats if/when knee pain is an issue). The THM will be covered extensively in this meeting, with a focus on application to exercise performance. For instance, after receiving an overview of the THM, participants will be guided in constructing their THM Recipes, which they can revise as needed throughout the intervention to find the best match for them personally. They will be told that they can always do more, and to feel free to perform with “High Effort” whenever they are motivated and able, but to focus on consistently performing the *tiny Behaviors* in their Recipes (e.g., 1 push-up) and celebrating each time they are successful. This approach aims to cultivate automaticity (i.e., habit formation) of the tiny behavior, thereby forming a stable habit base from which the target behavior (e.g., push-ups with High Effort) can be pursued when motivation is high. High Effort will be defined for participants as performing a set of exercise to within 1–2 repetitions of their Self-determined Repetition Maximum [52].

After the initial coach meeting described above, digital coaching will be utilized throughout the intervention phase. The PI will deliver the digital coaching, which will primarily be email-based, with daily contact during the first week and at least weekly contact thereafter. The Tiny Habits® Greenhouse will be utilized during the first week of the intervention phase for all participants, with the option to opt-in in subsequent weeks. The Greenhouse is a semi-automated, email-based system developed to allow Certified Tiny Habits® Coaches to deliver a five-day (Monday–Friday) program in which individuals receive daily feedback as they practice applying the THM. One-on-one videoconferencing will also be utilized on an as-needed basis throughout the intervention. Digital coaching will primarily focus on applying the THM and addressing barriers to boost adherence and engagement.

### Assessments

#### Primary outcome

The primary outcome of interest is the change in strength from baseline to 12-week follow-up, in the intervention group compared to the waitlist control group. Strength will be determined by the composite number of maximum push-ups, angled-rows, and bodyweight-squats that the participant can perform under a standardized protocol. All participants will perform angled-rows first, followed by bodyweight-squats, and then push-ups. Prior to performing each exercise, the participant will watch a ~ 1 min video demonstrating proper performance. The researcher will then give a live demonstration of the exercise. The participant will then perform the exercise at a cadence of 2-s for the eccentric portion and 2-s for the concentric portion of the exercise, resulting in 4-s repetitions. A video that alternates the announcement of “down” and “up” every two seconds and displays repetition-number will be used to keep participants in cadence and track the number of repetitions performed. The participant will perform each exercise until either 1. volitional muscle failure (i.e., they cannot physically do another repetition), 2. proper form is lost, or 3. they get off cadence by > 1 repetition.

For angled-rows, the suspension trainer will be set up on a door and the length of the straps will be adjusted for each participant such that the handles hang freely at navel-height. After performing angled-rows, the participant will be given approximately 3 min to rest. During this rest period, the participant will watch the instructional video for bodyweight-squats, and the researcher will give a live demonstration. The participant will then perform bodyweight-squats, followed by another 3-min rest period, during which the participant will be prepared to perform push-ups. A researcher will observe participants’ performance of each exercise, give feedback on form as needed, and document the number of repetitions performed and participant-reported repetitions-in-reserve [53]. In preparation for possible adverse events, such as a hypoglycemic event or a more serious cardiovascular event, an emergency protocol has been developed and will be utilized, as needed.

#### Secondary outcomes

The following secondary outcomes of interest will be assessed at baseline, 12-week follow-up, and 24-week follow-up, for all participants. These include BMI, blood pressure, fasting lipid panel, HbA1c, anthropometry (hip-to-waist ratio, waist-to-height ratio), body composition (fat and fat free mass), and muscle thickness of the mid-thigh. Participants’ height (baseline only) and weight will be measured with their shoes off while wearing only athletic clothing, and these measures will be used to calculate BMI. Blood pressure will be measured in both arms via an automated arm cuff system (Model BP785N, OMRON) following five minutes of rest, and the higher blood pressure will be utilized for data analysis. Fasting lipid panel will be obtained using the CardioCheck Plus Analyzer system (V1.09, PTS Diagnostics). To minimize the possibility of hypoglycemic events, participants will be provided a snack and drink after lipids are assessed, prior to their strength assessment. HbA1c will be obtained using the A1CNow + system (PTS Diagnostics). Body composition will be assessed using bioelectrical impedance spectroscopy (SFB7, Impedimed Inc.) with the participants in a supine position following five minutes of rest. Mid-thigh muscle thickness of the right leg quadriceps will be assessed via B-mode ultrasound (Logiq S7 R2 Expert; General Electric) at the mid-way point between the inguinal crease and proximal border of the patella. All ultrasound images will be collected with the participants in a standing position with body weight being placed on the left leg.

**Fig. 1:**
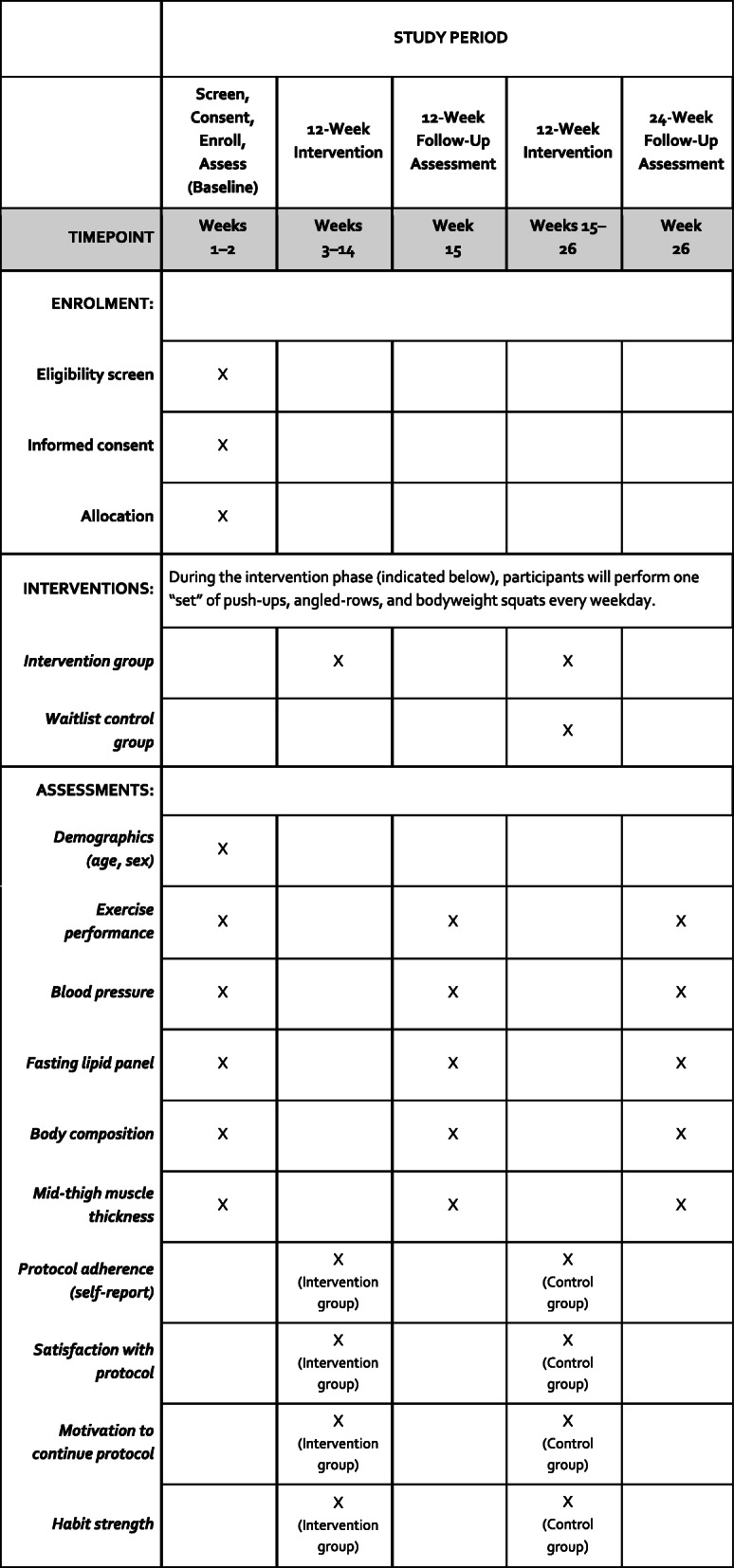
Relative timeline for enrollment, interventions, and assessments

Other secondary outcomes of interest will be assessed weekly via self-report over the initial 12 weeks of the intervention for all participants. These include adherence to the exercise protocol during the prior week, perceived exertion [53] when performing the exercises during the prior week, satisfaction with the protocol to date, motivation to continue the protocol in the coming week, confidence in their ability to adhere to the protocol in the coming week, and habit strength with regards to current THM Recipes and exercise performance, as measured by the Self-Report Behavioral Automaticity Scale [54]. A link to a Qualtrics-built survey containing these assessments will be emailed to participants each Friday during these 12 weeks. See Fig. 1 for a SPIRIT flow diagram regarding the relative timeline for enrollment, interventions, and assessments.

#### Investigators’ roles during assessments

SA and AA will assess blood pressure and screening forms, subsequently consent and enroll qualifying participants, and then assess fasting lipids and HbA1c. SA and AA will also assess blood pressure, fasting lipids and HbA1c at 12-week and 24-week follow up. KY and ZH will assess anthropometry, body composition, and muscle thickness of the mid-thigh at all three timepoints. JH and BE will perform strength assessments at baseline, prior to participant randomization, and BE will perform strength assessments at 12-week and 24-week follow-up. DW, who has a Master Degree in Exercise Physiology and a Doctorate Degree in Exercise Science, oversaw the development of the strength assessments as well as the participant training protocol.

### Statistical analysis

All statistical analyses will be conducted using SPSS software (v.25, IBM). The primary outcome of changes in strength between groups will be statistically evaluated using a one-way analysis of covariance (ANCOVA) using the baseline strength variable as the covariate. For secondary and exploratory analysis following 24 weeks of the intervention, a 2 × 2 mixed-factorial ANOVA with repeated measures [group (Control vs. Intervention) x time (pre vs. post)] will be used to analyze changes between groups and across time for each dependent variable. Significant interactions will be followed up using a bonferroni post hoc analysis. An alpha-level of *p* ≤ 0.05 will be used to determine statistical significance. All data will be presented as mean ± standard deviation (SD). KY will perform all statistical analysis, with blinding as to group assignment. Data will be analyzed at both 12 and 24 weeks for treatment effects, with 12-week data analyzed blindly. Unfortunately, due to the nature of the study design, 24-week data will be analyzed unblinded. All participants will be given a summary of the study results.

## Discussion

This study takes a unique approach to RT that is easily implemented and highly scalable. It aims to help participants add brief bodyweight exercises into their existing daily routine using a habit-based approach. Primary focus is placed on consistency and habit-formation first, with effort being increased as the participant is motivated and able. The approach requires little time, cost, and equipment, and can be performed nearly anywhere by individuals at nearly any fitness level, given proper training. The approach is flexible, with THM Recipes being revised as needed to find the best possible fit for the individual and their current daily routine.

This study could greatly inform and impact clinical practice. Regularly engaging in health behaviors, including RT, is a health promotion and disease prevention strategy that is considered first line treatment for nearly all chronic disease states. As such, RT of some form is recommended by the U.S. Physical Activity Guidelines for nearly all individuals. Thus, simple and effective approaches are needed to facilitate adoption and maintenance of RT. If the RT protocol in this study proves to be effective, it can be easily disseminated by healthcare providers, such as physicians, nurses, and health and wellness coaches who work with patients to facilitate health behavior change. As such, primary publication, as well as conference poster or presentation detailing the results will be pursued.

This study is not without limitations. Participants will not be supervised when they perform their exercises and will self-report their level of engagement by completing a weekly assessment sent via email. Additionally, it has been shown that the validity of repetitions-in-reserve as a measure of intensity is dependent upon one’s ability to accurately gauge their proximity to momentary muscular failure [55]. Thus, one limitation is the reliability of participant self-reporting in regards to adherence to the exercise protocol and the intensity with which they perform the exercises. However, having participants perform the exercises unsupervised is more representative of real-world implementation and strengthens external validity. That said, the use of financial incentives is a potential threat to external validity and may limit the applicability of findings to examining efficacy and not effectiveness of the intervention [56]. Another limitation is sample size. Although the study is powered to detect the expected increase in strength in the intervention group relative to the control group, having only 40–60 participants, all of who are either university/college office workers or osteopathic medical students, limits the extent to which results can be generalized. Additional studies will be needed to assess the effectiveness of the program in relation to other populations.

## Data Availability

Not applicable.

## Abbreviations

BMI: Body mass index
HbA1c: Hemoglobin A1c
HDL: High density lipoprotein
LDL: Low density lipoprotein
RT: Resistance training
THM: Tiny Habits® Method
U.S.: United States
